# Socio-economic, trafficking exposures and mental health symptoms of human trafficking returnees in Ethiopia: using a generalized structural equation modelling

**DOI:** 10.1186/s13033-018-0241-z

**Published:** 2018-10-24

**Authors:** Lemma Derseh Gezie, Alemayehu Worku Yalew, Yigzaw Kebede Gete, Telake Azale, Tilman Brand, Hajo Zeeb

**Affiliations:** 1 Department of Epidemiology and Biostatistics, Institute of Public Health, College of Medicine and Health Sciences, University of Gondar, Gondar, Ethiopia; 2 Leibniz Institute for Prevention Research and Epidemiology-BIPS, Bremen, Germany; 3 Department of Preventive Medicine, School of Public Health, College of Health Sciences, Addis Ababa University, Addis Ababa, Ethiopia; 4 Department of Health Education and Behavioral Sciences, Institute of Public Health, College of Medicine and Health Sciences, University of Gondar, Gondar, Ethiopia; 5 Health Sciences Bremen, University of Bremen, Bremen, Germany

**Keywords:** Human trafficking, Returnees, Mental health, Ethiopia

## Abstract

**Background:**

Mental health problems among trafficked persons could be the result of concomitantly interwoven effects of various factors. Analyzing the networked relationships concurrently could be a more substantive approach to better understand the role of risk factors in this population. This study aimed to assess the magnitude of mental health symptoms as well as the association among socio-demographic, trafficking related exposure variables, and mental health problems of Ethiopian returnees from trafficking.

**Methods:**

A sample of 1387 returnees who were trafficked via three major human trafficking corridors of Ethiopia were selected consecutively. Data related to socio-economic, trafficking exposure variables, and symptoms of mental illness were collected in personal interviews. Anxiety was measured with a brief measure for generalized anxiety disorder (GAD-7), depression with a patient health questionnaire (PHQ-9), and PTSD with post-traumatic checklist (PCL-C). Generalized structural equation modeling was employed to estimate the relationships among exogenous, mediating, and endogenous variables simultaneously.

**Results:**

The prevalence of symptoms of anxiety was estimated at 51.9% (95% CI 49.3–54.6%); PTSD was estimated at 34.5% (95% CI 32.1–37.1%) and depression at 58.3% (95% CI 55.6–60.9%). Restricted freedom of movement had a direct positive effect on anxiety (β = 1.24, 95% CI 0.97–1.51), depression (β = 0.94, 95% CI 0.71–1.17) and PTSD (13.00, 95% CI 11.23–14.77). Violence experienced during the trafficking period was a mediator variable and significantly associated with anxiety (β = 0.46; 95% CI 0.26–0.66) and PTSD (β = 4.00; 95% CI 2.06–5.94). History of detention had a positive total effect on GAD (total β = 1.380, 95% CI 1.074–1.687) and PTSD (total β = 15.63, 95% CI 13.708–17.545), and direct positive effect on depression (β = 0.89, 95% CI 0.65–1.13).

**Conclusion:**

Ethiopian trafficked persons were highly likely to return with increased levels of mental health symptoms, namely anxiety, depression, and PTSD. Socio-economic and trafficking related exposures mediated by violence were factors affecting mental health symptoms. Thus, in addition to economic re-integrations of victims, strategies should be designed and implemented to address the prevalent mental health problems.

**Electronic supplementary material:**

The online version of this article (10.1186/s13033-018-0241-z) contains supplementary material, which is available to authorized users.

## Background

Human trafficking, the requirement, transfer, and receipt of persons by means of deception or coercion for the purpose of exploitation [1], is a globally growing phenomenon despite the various efforts made by governments, local and international organizations [2]. The process of human trafficking involves individuals, families, and organized local or supra-regional criminal networks. The practice of luring individuals into such an exploitive condition is initiated and sustained by its lucrativeness at the expense of victims’ health and human rights. In Ethiopia, the practice of human trafficking is rampant [3] and trafficked persons are trapped in these network and transported to various parts of the world, mainly to the Middle East, Europe, and South Africa [4]. Despite a growing number of Ethiopian victims, minimum standards to address the problem of human trafficking are not met and the economic and social re-integration of returnees is insufficient [3, 4].

During the trafficking process, victims usually experience physical, sexual, or psychological violence by their traffickers. It is also common for them to get injured due to accidents or violence during travelling [5, 6]; they are also frequently exposed to infections such as malaria or STDs including HIV/AIDS. Women and girls may be exposed to unwanted pregnancy following forced or unsafe sexual practices during trafficking [7–10]. Moreover, they usually experience social exclusion and financial and labor exploitive conditions [11, 12]. As a result of all these problems and the high discrepancy between their expectations and the situation they encounter in reality, trafficked persons usually develop mental illness and other health problems, as was recently described for trafficked persons from Ethiopia [4].

According to the respective literature, anxiety among trafficked persons ranged from 10% [13] to 90.20% [14], depression from 12.5% [15] to 86.0% [14], and post-traumatic stress disorder (PTSD) from 13.4% [14] to 77% [16]. The high heterogeneities among the findings related to symptoms of mental health illnesses were also confirmed by a systematic review [5]. This could be partly due to the disparity of the study populations with respect to risk factors of mental illness, the specific situation that they were exposed to during the trafficking process, or other characteristics.

Depending on actual conditions during trafficking, the magnitude, and severity of mental health problems and its associated factors may vary by socio-demographic characteristics and trafficking exposures, including violence, time spent in a trafficking situation, type of exploitation, the extent of restriction of freedom and working hours, among others [11, 15, 16]. Because of contextual differences as a result of variations in all these factors and lacking information on the overall mental health of trafficked persons from Ethiopian, further studies with high quality methodology have been recommended [4]. The statistical analysis should take into account the interwoven relationships among the various risk factors and symptoms of mental illness, and direct and indirect pathways should be explored through the analysis.

However, our review of the literature revealed a dearth of studies in the topic especially in Ethiopia [4] and the available studies were based upon purposively selected localities and returnees [17]. This limits the generalizability or external validity of findings as there could be variation in the magnitude of mental health problems and their risk factors by socio-economic and trafficking exposures [11, 15, 16]. Moreover, studies on the symptoms of mental health problems of trafficked persons were analyzed with univariate analysis while advanced models that take into account the complex network of relationships among symptoms and risk factors are required.

In response to these identified gaps, we conducted a large study based on sufficient sample size with appropriate sampling procedure and employing a multivariate statistical analysis. Victims of trafficking who were returning to Ethiopia were intercepted at the three border cities via the three major human trafficking corridors until the required sample size was met. Trafficked persons from every corner of the country and not limited to a specific segment of the population of migrants were included. Our main objectives were (a) to determine the magnitude of symptoms of mental health illnesses, and (b) to examine the association among socio-economic, trafficking related exposure variables, and symptoms of mental health problems among Ethiopian returnees from trafficking. To account for the interdependency of various factors and mental health symptoms, generalized structural equation modeling was employed.

## Methods

### Study setting and period

The current investigation is embedded in a set of studies conducted with trafficking returnees in Ethiopia recruited between May 2016 and October 2016 [18, 19]. The focus of the current analysis was on the mental health experience of trafficked persons whereas an earlier analysis investigated physical violence [19]. Ethiopian returnees were recruited into the study while they were entering via three border towns, namely Moyale, Metemma-Yohannes, and Galafi. These towns are the major gates through which people from Ethiopia were trafficked. Moyale is a Southern border city in which people from Ethiopia are usually trafficked into South Africa via Kenya and other Southern African countries; Metemma-Yonannes is another border town in the North-West part of the country through which people are trafficked into Sudan, the Middle East, and Europe. Galafi is located in the North-East part of the country bordering Djibouti, and traffickers use it to transfer migrants to Yemen or the Middle East, mainly to Saudi Arabia. Trafficked persons who returned to their origin either on their own will or by deportation via these three border towns were included into the study irrespective of their exit corridor; few returnees used other corridors to go abroad, like Bole International Airport or Jigjiga, Humera, and Gambella corridors and included into the study as far as they entered via the three selected gates during return.

### Study design

A cross-sectional study design was employed to take a snapshot of population of returnees from trafficking over the 6-month period (May 2016–October 2016). Returnees were intercepted, recruited, and interviewed personally by trained interviewers just at the time of their entrance into Ethiopia. Thus, data related to the socio-economic, trafficking exposure, and mental health symptoms were collected from each returnee at a point in time during the specified period.

### Population, sampling procedure, and sample size

The study population consisted of Ethiopians who were trafficked trans-nationally and were returned via border corridors either on their own will or by deportation. As not all migrants are subjected to trafficking, their trafficking status was ascertained based on the definition of the UN 2000 protocol of human trafficking. If a returnee’s age was under 18, or if he/she was recruited for the purpose of exploitation or there was an evidence of exploitation (extended working hours, unpaid wage, sexual exploitation, etc.), then the returnee was considered as trafficked. Details about the screening and sampling procedure are available elsewhere [18].

A pilot study conducted on returnees showed that they could well recall the conditions and events they experienced during the preceding 2 years. Thus, Ethiopian victims of trafficking who left their country during the past 3–24 months and returned via the selected three major trafficking corridors were included in the study consecutively starting from May 2016 until the required sample size was met.

The adequacy of the sample to address the objectives set in this study using generalized structural equation modeling (GSEM) was checked based on the number of estimated parameters in the model; a 1:10 ratio of respondents to free parameters to be estimated has been recommended [20]. Accordingly, considering the 77 parameters to be estimated based on the hypothesized model, taking participants to free parameters ratio of 10, and a design effect of 1.5 (because we used cluster sampling of corridors), the required sample size was estimated to be 1271. Thus, the full sample of 1387 participants used for the analysis of physical violence [19] was also considered for the current analysis.

### Variables and measurement

**Fig. 1:**
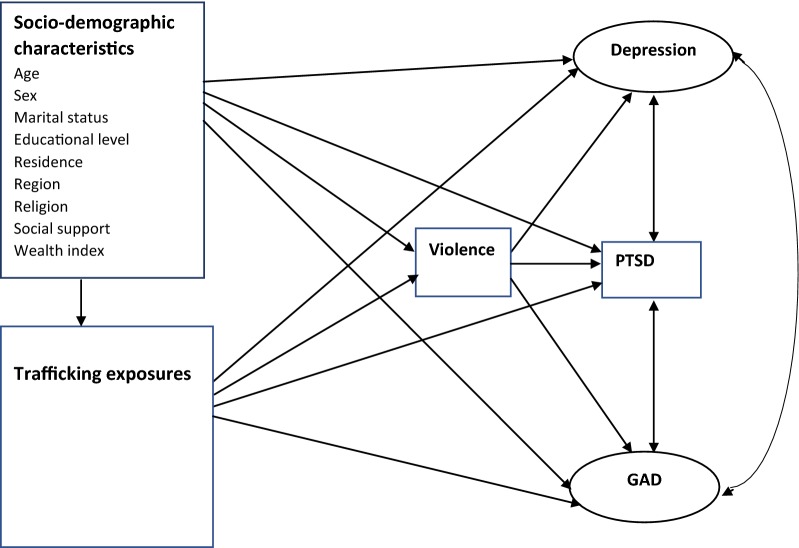
Hypothesized model for factors associated with mental health symptoms (depression, anxiety, and PTSD). *PTSD* post traumatic stress disorder, *GAD* general anxiety disorder; violence includes either physical, sexual or both types of violence; single headed arrows show direction of effect; double headed arrow shows correlation

Interview questions or variables covered current mental health symptoms as well as conditions well before departure and detailed aspects of situation during trafficking. Considering these variables, a hypothesized model based on literature about mental health symptoms among trafficked persons was formulated (Fig. 1). The model was developed in order to examine the effect of socio-demographic and trafficking related exposure variables on the mental health outcomes and the possible mediation by violence (physical, sexual, or both experienced during trafficking).

The mental health symptoms studied include anxiety, depression, and PTSD; they were endogenous variables measured with the respective scales. Anxiety and depression were continuous latent variables. Because the scale for PTSD had a large number of items (17 questions each with 5 response options), considering it as a latent variable increased the model complexity that took longer time to converge during analyses. To get a more parsimonious model, we conducted sensitivity analyses by comparing the fitness and simplicity of two competing models: a model when PTSD was taken as a continuous latent variable and another model when it was considered as a continuous observed variable; PTSD was changed into continuous observed variable by adding the score of item responses for each participant as described by the authors of the scale [21]. Thus, according to the result from the sensitivity analyses, considering PTSD as a continuous observed variable fitted the data well and gave us a simpler model as the number of parameters to be estimated would be reduced by the number of items in the scale.

The socio-demographic characteristics include age, sex, marital status, educational levels, residence, region, religion, social support, type of job at destination, and wealth quintile before trafficking. The trafficking related exposure variables were: restricted freedom of movement, smuggling status, substance use (concerning shisha, hashish and alcohol, etc.), history of detention abroad, and time since departure measured in months (Fig. 1).

Anxiety was assessed by using the generalized anxiety disorder tool (GAD-7). The seven items address specific symptoms of anxiety with 4 response options (‘Not at all’, ‘Several days’, ‘More than half of the days’, and ‘Nearly every day’) that were coded from 0 to 3 in order. Thus, the total score of anxiety for each study participant would range from 0 to 21, and a higher score indicates a higher degree of anxiety. The scale can be used in a dichotomized form where anxiety is considered as positive if the sum score is 10 or more. The score can also be transformed into a severity measure where a sum score of 4 or less is considered as no anxiety, 5–9 as mild, 10–14 as moderate, and 15 or more as severe symptom of anxiety [22].

Depression was measured with Patient Health Questionnaire (PHQ-9). The nine items in the instrument are ordinal indicators each with the same response options as in the GAD-7. The total score ranges from 0 to 27. In the dichotomized form of the scale, depression is considered as positive if the sum score is 10 or above; as a severity measure, a sum score of 0–4 would be considered as no depression, 5–9 as mild, 10–14 moderate, 15–19 moderately severe, and 20–27 severe depression [23]. Both anxiety and depression were assessed by considering the mental health experiences of returnees during the 2 weeks prior to the interview. On the other hand, symptoms of PTSD experienced during the past 1 month were measured with a civilian version of the PTSD Check List (PCL-C). It consists of 17 items each with five response options ranging from ‘Not at all’ or code ‘1’ to ‘Extremely’ or ‘5’. A sum score of 50 and above on the PCL-C scale supports presence of PTSD [21].

The scales were tested in various populations and the results found to be highly valid [21, 22], however, whether this also applies to populations of trafficked persons is unclear. In the current study, the Cronbach alpha reliability coefficient [24] for the GAD-7 anxiety scale was estimated at 0.914, for PHQ-9 depression scale at 0.928, and for the PCL-C PTSD scale at 0.960.

Physical and sexual violence were assessed based on a WHO international study of domestic violence [25]. Physical violence was assessed by asking whether returnees experienced specific acts including being kicked, dragged, beaten up, burned, being threatened with a weapon, cut with a knife, slaps, pushes, and hits. For women and girls, sexual violence was assessed by considering the three items of sexual acts: forced to have sex, had had sexual intercourse because of a fear if she was not compliant to it, or being forced to do something sexual that she found humiliating. Because the first two items related to sexual violence were considered very sensitive, we read all of them to each female respondent and asked whether she experienced any of these. Violence was scored positive if a person experienced at least one or any combinations of acts mentioned above under physical and sexual violence.

A composite wealth index was determined for the participants household using principal component analysis by weighting indexes generated from urban and rural specific items in the scale. There are 11 items in the tool, including household size, whether all children aged 6–12 years were attending school or not, and construction material of the walls of dwelling unit.

The Oslo three item social support scale was employed to assess returnees’ perception about the extent of care they would receive [26]. The sum score comprises valid values from 3 to 14 and was considered as a continuous variable for GSEM analysis. For descriptive purpose, a sum score ranging from 3 to 8 is classified as ‘poor support’, 9 to 11 as ‘intermediate support’, and 12 to 14 as ‘strong support’. Moreover, ‘restricted freedom of movement’ was scored positive if returnees had been locked in a house or a building or if respondents never had been free to go anywhere they wanted or do what they wanted [11].

### Data processing, model building, and analysis

The filled questionnaires were checked manually for completeness. Data were coded and entered into Epi-Info version 7 statistical packages and then exported to STATA version 14 for further analysis. Descriptive and summary statistics were done using figures and tables. The Generalized Structural Equation Modeling (GSEM) was employed to examine the relationship between various exogenous and endogenous or mediating variables. Because both anxiety and depression were latent variables which constitute items with ordered responses, their measurement model was analyzed with ordinal family and logit link function; PTSD was a continuous variable that was analyzed with gaussian family and identity link function, and violence was a binary outcome variable fitted with binomial family and logit link function. The analysis was started with the hypothesized model (Fig. 1), and modifications were performed iteratively by adding path links or including mediator variables, if theoretically supported, and comparing the information criteria of each model fitted. Finally, an over identified model with minimum information criteria was retained. The model was selected not only by considering the statistical significance of effects but also the theoretical meaningfulness of relationships, information criteria, and model parsimony. Diagrammatically, the effect of each exogenous or mediating variable on the respective dependent variable was indicated by the path coefficient along with single headed arrow, and the correlation among disturbances (residual errors that reflect the unexplained variances in the latent endogenous variables due to all unmeasured causes) was indicated by double arrows.

When mediation of effects was present, the direct, indirect, and total effects were determined using the non-linear combination of estimator technique. As there were few missing data for variables related to mental health symptoms, listwise deletion was employed in the GSEM analyses.

**Fig. 2:**
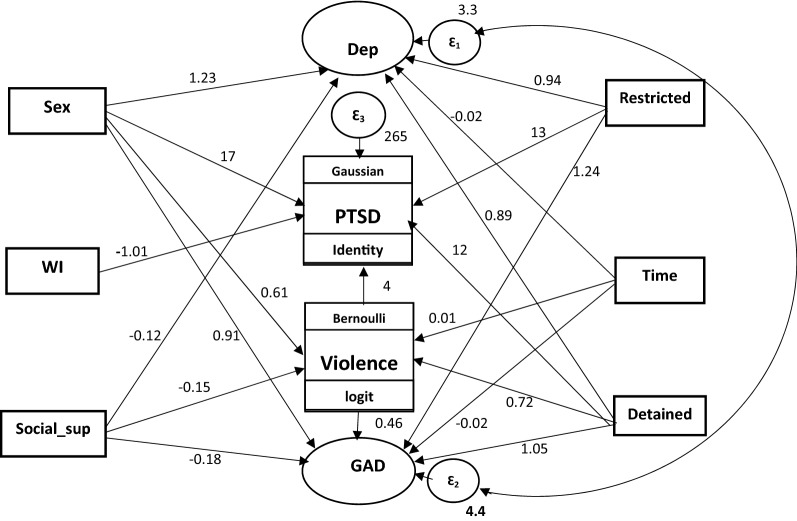
GSEM predicting mental health symptoms among Ethiopian trafficked persons, 2016 (structural model only). *Dep* depression, *PTSD* post-traumatic stress disorder, *GAD* generalized anxiety disorder, *WI* wealth index, *Social_sup* social support, *restricted* restricted freedom of movement, *time* time spent in trafficking situation, *detained* history of detention abroad by security personnel

The final model (Fig. 2) that fitted the data well and appeared theoretically meaningful was built by analyzing the hypothesized model (Fig. 1) and inspecting iteratively the statistical significance of path coefficients and the relevance of relationships in the model.

## Results

### Background characteristics

Of the 1525 trafficked persons who were approached for data collection, 1387 gave their consent to participate in the study [19]. Participants came from various socio-economic, demographic, and geographic backgrounds including some who were re-trafficked (i.e., repeatedly re-trafficked after returning to Ethiopia) indicating that the sample reflected the realistic picture of the situation of human trafficking among returnees in Ethiopia. Sociodemographic characteristics of interviewees are shown in Table 1. Slightly more than half of the participants were aged 21–25 years (50.1%), and over half (51.6%) of the participants were males. Over two-third of participants (70.22%) had poor social support (Table 1).

**Table 1:** Background characteristics of victims of trafficking from Ethiopia, 2016

Characteristics^a^	Number (percent) (n = 1387)
Age at departure	
14–17	84 (6.06)
18–20	420 (30.28)
21–25	695 (50.11)
26–49	188 (13.55)
Sex	
Male	716 (51.62)
Female	671 (48.38)
Marital status	
Married	267 (19.25)
Never married	1075 (77.51)
Separated	45 (3.24)
Educational level	
Illiterate	135 (9.73)
Primary	467 (33.67)
Junior	409 (29.49)
High school and above	376 (27.11)
Residence	
Rural	914 (65.90)
Urban	473 (34.10)
Region	
Oromyia	429 (30.93)
SNNP^b^	453 (32.66)
Amhara	345 (24.87)
Others	160 (11.54)
Religion	
Muslim	806 (58.11)
Protestant	342 (24.66)
Orthodox	216 (15.79)
Others	20 (1.44)
Social support	
Poor	974 (70.22)
Medium	274 (19.75)
Strong	139 (10.02)

^a^Socio-economic characteristics that describe situations before departure or at their origin

^b^SNNP is the region of Southern Nations and Nationalities and Peoples

### Mental health problems among returnees

The prevalence of anxiety symptoms was estimated at 51.9% (95% CI 49.3–54.6), PTSD was 34.5% (95% CI 32.1–37.1), and depression was at 58.3% (95% CI 55.6–60.9%). Whereas 17.5% of participants were screened as free of anxiety, 30.5% showed mild, 25.3% moderate, and 26.7% severe levels of anxiety. Only about 13% had no signs of depression, mild for 28.9%, moderate for 19.34%, moderately severe for 13.1%, and severe for 25.8% of the participants. Table 2 shows descriptive data on mental health symptoms for various characteristics, and the column number and descriptions relate to the cutoff points described above. Three-fourth (75.3%) of the participants who were trafficked via Galafi had symptoms of anxiety, 72.3% depression, and 70.4% had symptoms of PTSD. Many of the participants who were in a situation of restricted freedom of movement scored positive for symptoms of anxiety (61%), depression (65%), and PTSD (43%) (Table 2).

**Table 2:** Mental health symptoms by various characteristics of Ethiopian victims of trafficking, 2016

Characteristics	Number (percent)		
	Anxiety (n = 1369)	Depression (1370)	PTSD (n = 1372)
Age at departure			
14–17	41 (48.81)	50 (60.24)	12 (14.46)
18–20	222 (53.75)	236 (57.28)	134 (32.45)
21–25	360 (52.55)	402 (58.43)	271 (39.33)
26–49	89 (47.59)	109 (58.29)	54 (28.88)
Sex			
Male	336 (47.39)	362 (50.84)	205 (28.79)
Female	376 (56.97)	435 (66.11)	266 (40.30)
Residence			
Rural	481 (53.33)	518 (57.43)	333 (36.84)
Urban	231 (49.46)	279 (59.62)	138 (29.49)
Social support			
Poor	561 (58.13)	599 (62.07)	363 (37.58)
Medium	111 (41.57)	147 (54.85)	71 (26.39)
Strong	40 (29.20)	51 (37.23)	37 (27.01)
Smuggled at departure			
Yes	113 (40.50)	139 (49.47)	67 (23.59)
No	599 (54.95)	658 (60.42)	404 (37.13)
Trafficking corridor			
Metemma-Yohannes	348 (43.94)	428 (53.90)	161 (20.23)
Moyale	89 (45.18)	10 1 (51.27)	55 (27.92)
Galafi	265 (75.28)	254 (72.36)	247 (70.37)
Others	10 (35.71)	14 (50.0)	8 (28.57)
Type of job at destination			
Domestic work	288 (58.06)	335 (67.68)	186 (37.42)
Agriculture/animal farm	178 (47.09)	186 (49.34)	160 (42.55)
Manufacture and services	118 (47.97)	135 (54.88)	74 (30.08)
Did not start	108 (53.20)	115 (56.37)	39 (19.12)
Others	20 (43.48)	26 (54.17)	12 (24.49)
Restricted freedom			
Yes	503 (61.04)	540 (65.53)	355 (42.93)
No	209 (38.35)	257 (47.07)	116 (21.28)
Duration of detention (weeks)			
Not detained	230 (40.64)	290 (51.42)	117 (20.63)
≤ 1	70 (50.00)	83 (58.04)	31 (21.68)
2–4	250 (68.12)	253 (68.94)	207 (56.40)
5–12	78 (80.41)	74 (76.29)	64 (66.67)
13–51	84 (42.21)	97 (48.74)	52 (26.13)
Violence			
Yes	522 (59.79)	560 (64.29)	356 (40.83)
No	522 (52.01)	237 (47.49)	115 (23.00)

Violence = either physical, sexual or both types of violence experienced during the trafficking period; smuggled at departure = smuggling started to pass Ethiopian border and beyond but not smuggling started in other countries

For anxiety data, 18 persons had at least one missed score (n = 1369); similarly, for depression 17 persons (n = 1370), and for PTSD 15 persons had at least one missed score (n = 1372)

### Factors associated with symptoms of mental health problems

The final model containing only the structural part (relationships among latent or observed variables) is shown in Fig. 2 and Table 3. A model containing both the structural and measurement (relationship between a latent variable and its indicators or items) components is available in the additional file (Additional file 1). The fitted model was relatively parsimonious and had the minimum Akaike information criteria value when compared with other competing models. Several variables, namely age, marital status, education, residence, region, religion, type of job, substance use, and smuggling were excluded from the final model as their contributions were not statistically significant at an alpha level of 0.05.

**Table 3:** The direct, indirect, and total effects of socio-demographic and trafficking exposure characteristics on symptoms of mental health problem among Ethiopian victims of trafficking, derived from the GSEM, 2016

Characteristics	Direct effect (95% CI)	Indirect effect (95% CI)	Total effect (95% CI)
DV: violence (effect measure is OR)			
Sex			
Male	1.0		
Female	1.84 (1.47–2.29)	–	–
History of detention			
No	1.0		
Yes	2.06 (1.67–2.53)	–	–
Social support	0.86 (0.83–0.90)	–	–
Time in trafficking	1.01 (1.005–1.015)	–	–
DV: depression (effect measure is β)			
Sex			
Male	0.0		
Female	1.23 (0.98–1.49)	–	–
Restricted freedom			
No	0.0		
Yes	0.94 (0.71–1.17)	–	–
History of detention			
No	0.0		
Yes	0.89 (0.65–1.13)	–	–
Social support	− 0.12 (− 0.16 to − 0.08)	–	–
Time in trafficking	− 0.015 (− 0.02 to − 0.01)	–	–
DV: anxiety (effect measure is β)			
Violence			
No	0.0		
Yes	0.46 (0.26–0.66)	–	–
Sex			
Male	0.0	0.0	0.0
Female	0.91 (0.63–1.19)	0.280 (0.123–0.437)	1.190 (0.884–1.497)
Restricted freedom			
No	0.0		
Yes	1.24 (0.97–1.51)	–	–
History of detention			
No	0.0	0.0	0.0
Yes	1.05 (0.77–1.33)	0.332 (0.160–0.504)	1.380 (1.074–1.687)
Social support	− 0.18 (− 0.23 to − 0.13)	− 0.068 (− 0.103 to − 0.034)	− 0.2464 (− 0.309 to − 0.183)
Time in trafficking	− 0.016 (− 0.02 to − 0.01)	0.005 (0.002–0.008)	− 0.012 (− 0.018 to − 0.006)
DV: PTSD (effect measure is β)			
Violence			
No	0.0		
Yes	4.00 (2.06–5.94)	–	–
Sex			
Male	0.0	0.0	0.0
Female	16.72 (15.58–17.87)	2.434 (0.960–3.908)	19.157 (17.727–20.586)
Restricted freedom			
No	0.0		
Yes	13.00 (11.23–14.77)	–	–
History of detention			
No	0.0	0.0	
Yes	12.74 (11.14–14.34)	2.887 (1.255–4.518)	15.627 (13.708–17.545)
Wealth index	− 1.01 (− 1.57 to − 0.45)	–	–

*DV* dependent variable, *CI* confidence interval, *OR* odds ratio

The measure of association (effect measure) for violence was OR, and that of anxiety, depression and PTSD was β or beta; the βs are unstandardized coefficients

In the fitted model, all the path coefficients in the diagram were statistically significant at an alpha level of 0.05. Accordingly, the model included only six exogenous variables (sex, wealth index, social support, history of detention, restricted freedom of movement, and time spent in trafficking situation), one mediator variable (violence), and the three endogenous variables (GAD, PTSD, and Depression). Four exogenous variables, namely sex, social support, history of detention, and time in trafficking situation were both directly and indirectly related with GAD and PTSD via the mediator variable i.e. violence.

Specifically, violence has a direct positive effect on anxiety (adjusted β = 0.46, 95% CI 0.26–0.66). Among upstream variables (exogeneous variables related with the mediator variable, violence), female sex had both direct (adjusted β = 0.280, 95% CI 0.123–0.437) and indirect positive effects (adjusted β = 0.280, 95% CI 0.123–0.437) on anxiety that resulted in a positive total effect (adjusted β = 1.190, 95% CI 0.884–1.497). Social support had a negative direct (adjusted β = − 0.18, 95% CI − 0.23 to − 0.13) and indirect (adjusted β = − 0.068, 95% CI − 0.103 to − 0.034) effects, and accordingly a negative (i.e. protective) total effect on anxiety (adjusted β = − 0.2464, 95% CI − 0.309 to − 0.183). Similarly, history of detention had positive direct (adjusted β = 12.74, 95% CI 11.14–14.34) and total (adjusted β = 15.627, 95% CI 13.708–17.545) effects on PTSD (Table 3). The covariance between the disturbances for depression and anxiety was statistically significantly different from zero (covariance = 2.82, 95% CI 2.39–3.24) showing that there is strong correlation between the two mental health symptoms.

## Discussion

### Key findings

To the best of our knowledge, this is one of the largest studies done on the mental health of trafficked persons so far. Our findings demonstrated a high frequency of probable anxiety, depression, and PTSD symptoms among Ethiopian returnees. Violence was associated with anxiety and PTSD; it was also a significant mediator variable between these two mental health problems and most of the exogenous variables in the model that were either socio-economic or trafficking exposures. However, for depression we saw only direct effects for most of the exogenous variables in the model.

In this study, about half of the returnees had symptom of anxiety, almost one-third showed PTSD symptoms, and over half of them reported depression. Our estimate of general anxiety disorder was much greater than the prevalence of anxiety disorder for the general population in Ethiopian (3.3%) [27] and that of the Syrian post war refugees (31.3%) [28], but comparable with some studies conducted on trafficked persons [11, 29]. Of course, high variation in the magnitude of anxiety among trafficked persons has been reported in the literature, ranging from about 6% [15] to 90% [14]; similar variations were observed for depression and PTSD. The high rate of mental health symptoms identified at the end of the trafficking period could be explained by the extreme forms of abusive conditions respondents experienced during the trafficking periods that usually involve strict control, violence (physical, sexual, and psychological), torture (that could happen sometimes to get money from the family of trafficking victims), intimidations, deprivations and social stigma [30, 31]. Pre-trafficking conditions like personality factors, childhood conditions, and socio-economic status could also contribute to the high level of mental health problems [32].

The high heterogeneity of the prevalence of the mental health symptoms reported by previous studies could also be related with the variation in these predisposing factors among the respective study populations considered. Moreover, the time point during the trafficking process at which the assessment of symptoms was conducted also is likely to be another factor influencing results across studies. For instance, our study assessed anxiety and depression during the last 2 weeks prior to the interview at the port of return to Ethiopia, and PTSD during the last 1 month of the trafficking period. As some of the returnees were freed from the trafficking condition close to the date of interview, the rate of symptoms could be higher than in studies conducted long after the integration/reintegration or late during the post-trafficking period [29].

Mental health is a complex phenomenon [33] and our study addressed this situation by exploring and analyzing concomitantly the mental health outcome variables, violence experienced during trafficking [12], and other potential predisposing factors (socio-demographic and factors representing situations during trafficking) [11, 12, 16, 29]. Thus, this study is also unique in that the complex network of relationships among the factors that affect mental health of trafficked persons was also described by employing appropriate statistical technique.

Violence (physical, sexual, or both) experienced during trafficking was positively associated with anxiety and PTSD and this is of course in line with other reports [12, 34]. The abuse, intimidation, and mistreatment of migrants could result in stress that may in turn result in various mental health problems including anxiety. As a mediator variable, violence was positively associated with female sex, history of detention, and time in trafficking situation but negatively with social support.

The association between history of detention and violence could probably be related with the extra force used by detainers or the mistreatment during their detention at the transit or destination countries [35]. Time in trafficking situation is also positively associated with violence and this may not be surprising because as the duration of time spent in trafficking situation increases, the risk of experiencing violence also increases [14]. The negative association, i.e. a protective effect of social support before departure and violence could be probably explained by the support from families or others from the social network which might have continued even during the hard times after departure. For example, sometimes traffickers torture their victims to obtain money from their families, and in this extremely stressful situation, strong social support may play an important role to reduce the adverse influence of this serious human rights violation [36].

Socio-demographic variables (sex and social support) and trafficking related variables (restricted freedom, time in trafficking, and history of detention) predicted directly and independently both anxiety and depression; except restricted freedom, all of these variables were also indirect predictors of anxiety through violence as mediator. Studies showed that females are disproportionately affected by common mental disorders than males [37–39] due to gender specific risk factors like gender-based violence, low socio-economic status, and constant responsibility of caring for others [40, 41]. The situation could be worsening for females under trafficking condition, a severe form of violence. The high prevalence of physical and sexual violence to which females are exposed [29] and the correspondingly high rate of PTSD following such violence renders trafficked females the largest single group of people affected by this disorder.

Restricted freedom of movement has been shown to be an independent predictor of anxiety, depression [11, 12], and PTSD [11], probably linked to the stressful conditions such as strict control exerted over trafficked persons that limit their right to behave and move freely. Returnees who had better social support also had a reduced risk of developing symptoms of mental health. As the history of detention encountered during trafficking period increases, the risk of developing anxiety was also high. Some results supporting our findings have also been reported from women detained by police in Vietnam [34].

Interestingly, individuals who had stayed longer in trafficking situations were less likely to report symptoms of anxiety and depression during the last 2 weeks of their trafficking period. This contradicts with a finding from a study conducted among females in seven post-trafficking support services that reported a positive association between time in trafficking and anxiety or depression [16]. Of course, there are also studies reported inconclusive findings for anxiety, depression and PTSD [16, 34] and mental health disorders [15]. Here the association was between the time spent in trafficking situation and the presence of symptoms within the last 2 weeks of the respective trafficking period, but not with the presence of symptoms in the trafficking period overall. It is unclear whether adaptation to the exploitive conditions plays a role in this context. For example, the initial phases of trafficking could be particularly tragic and stressful for newly trafficked persons until they adapt—to some extent—to cultural and social disparities and other circumstances that could influence mental health. Individuals who developed symptoms of mental health problems early-on could recover from it during the trafficking period even without proper treatment [42]; and this could result in a negative association with time spent in trafficking situation. However, differences between studied populations in terms of sex, time covered by the interview questions etc. need to be considered when interpreting findings from this and other studies [16].

The presence of PTSD during the past 1 month was associated with socio-demographic variables (positively with female sex and negatively with wealth index) and trafficking exposures (positively for both restricted freedom and history of detention). Both restricted freedom of movement during traveling and history of detention by security personnel create stressful conditions increasing the frequency of PTSD, as our study shows, and again this may be true particularly for the disadvantaged group of women and girls [39, 40]. The household wealth index was negatively associated with PTSD. One possible explanation is that the financial and material capacity of families is to some extent protective against the strict control of traffickers [36] and eventually against subsequent PTSD symptoms.

### Strengths and limitations of the study

The current study used standardized questionnaire, and data were collected by trained and experienced field workers with close and supportive supervisions. For ethical reasons, participants who were severely mentally ill and unable to give information were excluded from the study. However, as the number of excluded persons was small, this might not substantially affect the magnitude and direction of effects. Generally, respondents were informed about the importance of the study and the confidentiality of personal data to gain the trust of respondents and minimize the nonresponse rate. As the assessment of mental illnesses was conducted when respondents just entering into Ethiopia from abroad, the PTSD assessed might not actually reflect the stress disorder during post-trafficking or traumatic period but rather acute stress disorder during the traumatic or trafficking period. Of course, this could be true for individuals who escape an exploitive condition and returned within a time shorter than 1 month as PCL-C captures mental health symptoms during the past 1 month only. The other limitation of the study is the use of assessment tools of mental illness but not diagnostic tools. A further limitation is the use of mental health assessment tools that have not been tested in specific populations such as trafficked persons. However, the validity and reliability of these tools were checked in the general population of culturally diversified contexts. We also found that the reliability of the scales used were high for all the three tools. On the other hand, as the study included only returnees, the findings might not be generalizable to those trafficked Ethiopians who are still outside the country, possibly in a continuous trafficking situation. However, we assume that our results are informative and valid with respect to the general population of returnees who experienced trafficking. Overall this is a highly sensitive research topic and population where high quality empirical research is a particular challenge.

### Implications

The high prevalence of mental illness could suggest that victims of trafficking should be provided with comprehensive reintegration and protective services, including psycho-social counselling and mental health treatments in addition to economic and legal assistance. Moreover, as the employed tools screen mental illness within the most recent and fixed duration (for example, GAD-7 and PHQ-9 assess the presence of symptoms during the last 2 weeks and PCL-C during the last 1 month), similar studies should be conducted at each stage of the human trafficking process so as to understand fully the pattern of symptoms over time. This is related with the concern that the distribution of predisposing factors for mental illness overtime along the stages of human trafficking could vary according to the conditions victims underwent in those traumatic periods. It is also worth employing clinical diagnostic procedures to examine mental health symptoms of trafficked persons, while the core responsibility undoubtedly lies in preventing the human rights violation of human trafficking. The long-term mental health consequences in this segment of the population with a high burden of mental health problems after their return to Ethiopia should also be examined.

## Conclusion

Ethiopian trafficked persons were highly likely to return with increased levels of mental health problems, namely anxiety, depression, and PTSD. Socio-demographic factors (sex, wealth index, social support) and trafficking related exposure variables (time spent in trafficking situation, restricted freedom, and history of detention) mediated by violence (physical, sexual, or both) were factors associated with mental health status. Thus, in addition to the economic reintegration of victims, strategies should be designed and implemented to address the prevalent mental health problems among returnees.

## Additional file

**Additional file 1.** GSEM predicting mental health symptoms among Ethiopian trafficked persons, 2016 (both structural and measurement models).
