# Supplementary material for: Socio-economic, trafficking exposures and mental health symptoms of human trafficking returnees in Ethiopia: using a generalized structural equation modelling
Source: Int J Ment Health Syst. 2018 Oct 24;12:62. doi: 10.1186/s13033-018-0241-z (PMC6199759; doi:10.1186/s13033-018-0241-z)
Supplement: Supplementary file 1 — Additional file 1. GSEM predicting mental health symptoms among Ethiopian trafficked persons, 2016 (both structural and measurement models). [file 13033_2018_241_MOESM1_ESM.docx]

**Figure: GSEM predicting mental health symptoms among Ethiopian trafficked persons, 2016 (both structural and measurement models)**

Dep=depression; PTSD=post-traumatic stress disorder; GAD=generalized anxiety disorder; WI=wealth index; Social_sup = social support; Restricted= restricted freedom of movement; Time=time spent in trafficking situation; Detained= history of detention abroad by security personnel; a1-a7 are the seven indicators for the general anxiety scale used, d1-d9 are the nine indicators for depression scale used; ordinal family and logit link function were used in analyzing the ordinal anxiety and Depression outcome variables; Bernoulli family and logit link function were used in analyzing violence, a binary outcome variable; gaussian family and identity link functions were employed to analyze the continuous PTSD outcome variable
